# Supplementary material for: Altered carnitine-acylcarnitine profiles in levothyroxine-treated congenital hypothyroid patients with fatigue: An LC-MS/MS-based study from Bangladesh
Source: PLoS One. 2025 Sep 25;20(9):e0331474. doi: 10.1371/journal.pone.0331474 (PMC12463203; doi:10.1371/journal.pone.0331474)
Supplement: S1 Table — (DOCX) [file pone.0331474.s001.docx]

**Table S1.** **Adjusted p-values and corresponding power of the significant parameters from Table 3**

| **Parameters** | **Patient** | **Healthy Control** | **p-value** | **p-value†** | **Power** |
| --- | --- | --- | --- | --- | --- |
|  | µmol/L | µmol/L |  |  |  |
|  | (mean±SD) | (mean±SD) |  |  |  |
| Free Carnitine (C0) | 45.38±12.61 | 41.54±9.85 | 0.049 | 0.16 | 61.15 |
| Palmitoylcarnitine (C16) | 1.02±0.32 | 1.24±0.43 | 0.0003 | 0.037 | 85.81 |
| Stearoylcarnitine (C18) | 0.39±0.14 | 0.47±0.17 | 0.002 | 0.16 | 58.07 |
| Oleylcarnitine (C18:1) | 0.7±0.21 | 0.81±0.25 | 0.007 | 0.27 | 43.15 |
| Hydroxypalmitoylcarnitine (C16OH) | 0.001±0.002 | 0.01±0.003 | 0.001 | <0.01 | 96.38 |
| 3-Hydroxystearoylcarnitine (C18-OH) | 0.001±0.003 | 0.001±0.003 | 0.013 | 0.049 | 80.61 |
| C0/C16+C18 | 34.55 ± 14.88 | 25.73 ± 6.87 | <0.001 | <0.01 | 98.69 |

† p-value adjusted for false discovery rate
