# Supplementary material for: Altered carnitine-acylcarnitine profiles in levothyroxine-treated congenital hypothyroid patients with fatigue: An LC-MS/MS-based study from Bangladesh
Source: PLoS One. 2025 Sep 25;20(9):e0331474. doi: 10.1371/journal.pone.0331474 (PMC12463203; doi:10.1371/journal.pone.0331474)
Supplement: S2 Table — (DOCX) [file pone.0331474.s002.docx]

**Table S2. Comparison of 8 short chain and 7 medium chain acylcarnitines between patients and healthy controls**

| **Category** | **Patient**  **µmol/L**  **(mean±SD)** | **CV**  **(%)** | **IQR** | **Healthy Control**  **µmol/L**  **(mean±SD)** | **CV**  **(%)** | **IQR** | **P Value** |
| --- | --- | --- | --- | --- | --- | --- | --- |
| **Short chain acylcarnitines**  Acetylcarnitine (C2)  Propionylcarnitine (C3)  Butyrylcarnitine (C4)  3-Hydroxybutyrylcarnitine (C4OH)  Isovalerylcarnitine (C5)  Tiglylcarnitine (C5:1)  3-OH isovalerylcarnitine (C5OH*)* Glutarylcarnitine (C5DC)Total short chain acylcarnitines | 16.56±6.5  1.52±0.59  0.14±0.06  0.09±0.06  0.13±0.07  0.01±0.004  0.25±0.08  0.08±0.04  18.77±6.95 | 39.26  38.91  44.08  68.0  56.26  68.24  34.06  48.73  37.07 | 8.72  0.98  0.06  0.07  0.06  0.007  0.1  0.05  10.19 | 15.30±4.52  1.38±0.43  0.13±0.05  0.09±0.05  0.14±0.05  0.01±0.005  0.3±0.08  0.07±0.02  17.40±4.84 | 29.54  30.91  42.74  60.82  36.72  78.21  28.6  39.97  27.85 | 4.9  0.51  0.05  0.05  0.67  0.006  0.13  0.04  5.13 | 0.19  0.11  0.25  0.53  0.67  0.61  0.0012^*^  0.22  0.195 |
| **Medium chain acylcarnitines**  Hexanoylcarnitine (C6)  Octanoylcarnitine (C8) Octenoylcarnitine (C8:1) Decanoylcarnitine (C10) Decenoylcarnitine (C10:1) Decadienoylcarnitine (C10:2) Lauroylcarnitine (C12)Total medium chain acylcarnitines | 0.03±0.01  0.02±0.01  0.19±0.13  0.08±0.05  0.09±0.05  0.02±0.01  0.05±0.02  0.47±0.26 | 69.14  132.3  68.49  67.85  70.15  64.78  50.05  55.44 | 0.02  0.04  0.18  0.06  0.06  0.01  0.03  0.31 | 0.02±0.01  0.02±0.02  0.16±0.09  0.07±0.04  0.07±0.03  0.02±0.08  0.05±0.02  0.40±0.16 | 97.17  194.5  54.33  59.97  52.5  51.6  41.74  39.38 | 0.03  0.02  0.1  0.04  0.04  0.01  0.02  0.18 | 0.054  0.22  0.14  0.24  0.094  0.66  0.42  0.092 |
