# Supplementary material for: Altered carnitine-acylcarnitine profiles in levothyroxine-treated congenital hypothyroid patients with fatigue: An LC-MS/MS-based study from Bangladesh
Source: PLoS One. 2025 Sep 25;20(9):e0331474. doi: 10.1371/journal.pone.0331474 (PMC12463203; doi:10.1371/journal.pone.0331474)
Supplement: Attachment — Submitted filename: Responses to Reviwers.docx [file pone.0331474.s004.docx]

**Responses to the Reviewers’ Comments**

We sincerely thank all three reviewers for their thoughtful and constructive feedback. We have carefully revised the manuscript accordingly to improve its clarity, scientific rigor, and clinical relevance. Our detailed point-by-point responses are provided below.

**Reviewer #1**

Comment 1: The manuscript provides a descriptive account of the results but lacks sufficient emphasis on the broader clinical significance of the findings. The authors should expand the discussion to contextualize how these metabolic alterations may influence patient outcomes, management strategies, or future diagnostic considerations.

Response 1: We appreciate this important suggestion. We have now revised the discussion section and also included the below text in the conclusions;

“This study reveals significant metabolic alterations in congenital hypothyroid patients, including reduced long-chain acylcarnitine levels and inferred low CPT-I activity, suggesting impaired β-oxidation and energy metabolism. These disruptions may contribute to persistent fatigue, even in patients with normalized thyroid hormone levels under LT4 therapy. The findings highlight a critical gap in current management strategies that rely solely on hormonal normalization, underscoring the need for more comprehensive care that addresses underlying metabolic dysfunction.

Clinically, these insights suggest that routine metabolic profiling—especially of carnitine-acylcarnitine pathways—could serve as a valuable adjunct in assessing treatment response and guiding individualized therapy. Additionally, these results open avenues for considering adjunct interventions such as carnitine supplementation in selected cases. Future studies, particularly longitudinal and mechanistic investigations, are essential to validate these findings and refine diagnostic and therapeutic approaches for optimizing outcomes in congenital hypothyroidism.”

Comment 2: Clarify the interpretation of statistical significance, biological relevance, limitations (e.g., cohort heterogeneity, missing pre-treatment FT4 data), and expand on mechanisms and implications.

Response 2: We have revised the Results and Discussion sections to clarify statistical interpretations. In the Discussion section, we have included the limitations as

“This study provides valuable insights into metabolic disturbances in congenital hypothyroidism; however, several factors should be considered when interpreting the findings. The cross-sectional design limited our ability to track treatment outcomes over time, and fatigue was self-reported without standardized severity scoring. While triglyceride levels were assessed, additional metabolic or hepatic markers, including pre-treatment FT4 and urinary carnitine/acylcarnitine levels, were not available. Tissue-specific effects were also beyond the scope of this analysis. Additionally, variability in patient genetics and treatment history may have influenced the observed metabolic profiles.”

**Reviewer #2**

Comment 1: Statistical Power and Interpretation: Include power calculations to justify sample size. Clarify whether multiple testing correction was applied.

Response 1: Thank you for this important comment. As this was a cross-sectional study, we recruited patients from Bangabandhu Sheikh Mujib Medical University (BSMMU) over a period of three years, with sample collection spanning from 2016 to 2019. Congenital Hypothyroidism (CH) has a global incidence of approximately 1 in 3,000–4,000 children. In our study, we enrolled 56 participants under the age of 18, which reflects a representative subset of approximately 115,000 children in Bangladesh.

The below text is included in the methods section 2.2.1

“Patient recruitment was conducted twice weekly at the Pediatric Endocrinology OPD at BSMMU and National Institute of Nuclear Medicine and Allied Sciences (NINMAS), where around 50–60 children present daily with various endocrine disorders. Among them, only 1–2 were confirmed CH cases coming for follow-up. Most of these patients were late-diagnosed and under ongoing treatment with Levothyroxine (LT4)”.

While we recognize the value of a larger sample size, increasing enrollment was constrained by the limited frequency of CH cases and the scope of our study period. Given the rarity of CH and the logistical challenges involved, we believe our sample size represents a meaningful and substantial effort in the context of Bangladesh.

To address concerns regarding statistical power, we conducted post hoc power analyses. For the study's primary indicator—the C0/(C16+C18) ratio, which reflects a key metabolic pathway—we observed a power exceeding 90%, indicating a high likelihood of detecting true effects (please refer to Supplementary Table S1).

In addition, we have provided power estimates for all parameters that reached statistical significance. These power values are included in the supplementary file to aid interpretation. Regarding the risk of false positives due to multiple comparisons, we applied false discovery rate (FDR) correction to all statistically significant parameters. The adjusted p-values, alongside the corresponding power estimates, are also presented in the supplementary file for readers who wish to explore these results in greater detail.

Comment 2: Study Design and Cohort Matching: Clarify BMI matching and address other confounders such as diet, physical activity, and comorbidities.

Response 2: We sincerely thank the reviewer for this thoughtful observation. While the mean BMI in the patient group (17.0 ± 4.4) was slightly higher than that of the control group (15.78 ± 2.62), this difference was not statistically significant. Matching for age, sex, and BMI was conducted at the group level, based on the available demographic information.

We fully agree that additional covariates—such as dietary intake, physical activity, and comorbidities (e.g., liver dysfunction)—can influence metabolite levels and are important considerations in metabolic studies. Unfortunately, these data were not available in the present study. We have acknowledged this as a limitation in the discussion section and appreciate the reviewer’s attention to this important point.

Comment 3: Assay Validation and Consistency: Include key LC-MS/MS validation metrics and explain batch processing.

Response 3: We thank the reviewer for this insightful comment. In response, we have revised the Method Validation Section 2.5.

“The LC-MS/MS method used in this study was validated on the Shimadzu LCMS-8050 system (Shimadzu Corporation, Kyoto, Japan) using the NeoMass AAAC kit (Labsystems Diagnostics Oy, Vantaa, Finland). Method validation was performed in accordance with Clinical and Laboratory Standards Institute (CLSI) guidelines (EP5-A2, EP06, and EP17) to ensure the reliability and accuracy of quantification of amino acids and acylcarnitines from dried blood spot (DBS) specimens (12).

Validation was carried out using three levels of quality control (QC) DBS materials—low, medium, and high—provided with the NeoMass AAAC kit. The following parameters were evaluated:

Linearity: All analytes demonstrated strong linearity across the measurement range with coefficient of determination (R²) values >0.99.

Limit of Detection (LOD) and Limit of Quantitation (LOQ): LOD and LOQ were calculated for each analyte according to CLSI EP17 guidelines. The detailed values are presented in Supplementary Table S4.

Precision:

Intra-assay Coefficients of Variation (%CV): The intra-assay CVs for most amino acids and acylcarnitines were within 20%, except for C6 (36.47%) due to its low abundance in the QC sample (0.13 µmol/L).

Inter-assay Coefficients of Variation (%CV): Ranged between 1.32% and 11.60% for amino acids, and 1.16% to 14.14% for acylcarnitines, well within acceptable limits.

Accuracy: Assessed as relative error (RE%), which ranged from -19.85% to +9.33% for intra-assay and -19.31% to +3.55% for inter-assay measurements.

Recovery: Recovery rates for amino acids ranged from 80.68% to 103.54%, and for acylcarnitines from 93.37% to 108.35%, confirming acceptable performance.

To reduce potential batch effects, samples from healthy controls and clinically suspected patients were randomized and analyzed across multiple batches. Each LC-MS/MS run included low, medium, and high QC controls, ensuring assay performance was continuously monitored and remained consistent throughout the study period.”

These validation results confirm that the LC-MS/MS method is robust, accurate, and suitable for the quantification of targeted metabolites in DBS specimens.

We have added a brief summary of LC-MS/MS assay performance in the Methods section, including LOD, LOQ, and inter-assay CVs for major analytes. Samples were randomized across analytical batches, and internal standards were used to control for batch variation.

Comment 4: Interpretation of CPT-I Activity: The use of C0/(C16+C18) as a CPT-I proxy is indirect. Interpret cautiously and consider other explanations.

Response 4: We fully agree. The revised Result (3.3) and Discussion now acknowledges that C0/(C16+C18) is an indirect marker of CPT-I activity and may be influenced by transport or renal mechanisms. We interpret the findings more cautiously and suggest future studies with direct enzymatic assays or flux analysis to validate CPT-I dysfunction.

As CPT-1 activity was assessed indirectly in this study, we have revised the manuscript title to: “Altered Carnitine-Acylcarnitine Profiles in Levothyroxine-Treated Congenital Hypothyroid Patients with Fatigue: An LC-MS/MS-Based Study from Bangladesh”.

Comment 5: Plasma TG as a marker of hepatic FA metabolism should be interpreted cautiously without liver markers.

Response 5: We appreciate this observation and have moderated our interpretation of plasma triglyceride elevation. While the data suggest disrupted fatty acid metabolism, we acknowledge that hepatic TG accumulation cannot be confirmed without liver-specific data. However, we had some patient’s data on liver scan and found fatty liver which was not included in the manuscript.

Minor Points

Comment 1: Grammar and formatting issues.

Response 6: We have carefully revised the manuscript for grammar, punctuation, and formatting issues throughout.

Comment 2: Clarify the impact of missing FT4 baseline data.

Response 7: We have added a statement in the Limitations section acknowledging that the absence of pre-treatment FT4 values limits our ability to assess the association between baseline thyroid hormone levels and subsequent metabolic profiles.

Comment 3: Consider re-plotting with boxplots and individual data points.

Response 8: We have updated the Figure 1 in result section 3.3.

**Reviewer #3**

Comment 1: Expand the Limitations section to address the cross-sectional nature of the study, lack of follow-up, and absence of direct fatigue severity measures.

Response 1: Thank you. We have added these points to the Limitations section. The cross-sectional design precludes causal inference, and we acknowledge the lack of standardized fatigue scales. We propose that future studies incorporate longitudinal follow-up and validated fatigue assessments. We have included the below information in the discussion section;

“This study provides valuable insights into metabolic disturbances in congenital hypothyroidism; however, several factors should be considered when interpreting the findings. The cross-sectional design limited our ability to track treatment outcomes over time, and fatigue was self-reported without standardized severity scoring. While triglyceride levels were assessed, additional metabolic or hepatic markers, including pre-treatment FT4 and urinary carnitine/acylcarnitine levels, were not available. Tissue-specific effects were also beyond the scope of this analysis. Additionally, variability in patient genetics and treatment history may have influenced the observed metabolic profiles”.

Comment 2: Explicitly address the absence of urinary carnitine/acylcarnitine data.

Response 2: We have expanded the Limitations section to note that urinary profiling would have provided insight into renal carnitine handling and excretion, and that plasma-only data may not fully reflect systemic carnitine metabolism.

We again thank the reviewers for their thoughtful feedback and believe the revisions have substantially strengthened the manuscript. All changes have been highlighted in the revised version for clarity.
