# Supplementary material for: Altered carnitine-acylcarnitine profiles in levothyroxine-treated congenital hypothyroid patients with fatigue: An LC-MS/MS-based study from Bangladesh
Source: PLoS One. 2025 Sep 25;20(9):e0331474. doi: 10.1371/journal.pone.0331474 (PMC12463203; doi:10.1371/journal.pone.0331474)
Supplement: Attachment — Submitted filename: Response to Reviwers.docx [file pone.0331474.s005.docx]

**Responses to the Reviewers’ Comments**

We sincerely thank all reviewers for their thoughtful and constructive feedback. We have carefully revised the manuscript. Our detailed point-by-point responses are provided below.

**Reviewer #2:**

**Comment:** Thank you for this thoughtful and carefully revised manuscript. It’s clear that you’ve put in a lot of effort to address the reviewers’ concerns, and the improvements are noticeable throughout the paper. The addition of assay validation metrics, power calculations, and multiple testing corrections really strengthens the rigor of the study. I also appreciate the more cautious interpretation of the C0/(C16+C18) ratio as a proxy for CPT-I activity—your revisions make it clear that you’re aware of the limits of cross-sectional data, and you’ve acknowledged those thoughtfully.

The framing around persistent fatigue in LT4-treated patients is compelling, especially given how underexplored this topic is in the literature. The revised discussion nicely bridges the biochemical findings with potential clinical relevance, without overstepping the data.

A few suggestions for further tightening: Maybe try to avoid implying causality when discussing fatigue and metabolic changes—phrasing like “may contribute to” or “is associated with” works better given the study design.

If the reference to TPO and TSHR gene mutations comes from data in this cohort, a brief clarification would help. If not, it might be worth softening the phrasing to avoid overextending the findings.

Consistently referring to the patient group as “congenital hypothyroid patients” could help with clarity, especially for readers outside the endocrinology field.

Overall, this is a solid and well-executed study that adds something new to the conversation. I enjoyed reading it and appreciate the care you’ve taken in refining it.

**Response:** Thank you for your positive feedback and the careful reading of our revised manuscript. We have made the following specific changes in response to your suggestions:

**1. Softened causal language around fatigue and metabolic changes**

**Some** edits have been applied throughout the Abstract and Conclusion to replace phrasing such as “may contribute to” or “are associated with,” avoiding any implication of direct causality given our cross‐sectional design.

**Response:** We have revised the manuscript accordingly.

**2. Clarified the origin of** **TPO and TSHR mutation data**

**Response: The TPO and TSHR mutation data derived from the same cohort which is already published and we have clarified in the revised manuscript.**

**3. Standardized terminology to “congenital hypothyroid patients”**

**Response:** We have changed patient group as “congenital hypothyroid patients” for appropriate readability.
